# Screening for dementia and associated factors in older adults from low socioeconomic communities in iLembe, KwaZulu-Natal

**DOI:** 10.4102/hsag.v29i0.2437

**Published:** 2024-04-12

**Authors:** Xoli P. Mfene, Basil J. Pillay

**Affiliations:** 1 Department of Psychology, Faculty of Applied Human Sciences, University of KwaZulu Natal, Pietermaritzburg, South Africa; 2 Department of Behavioural Medicine, Faculty of Nursing and Public Health, University of KwaZulu Natal, Durban, South Africa

**Keywords:** dementia, epidemiology, low-middle income households, major neurocognitive disorders, prevalence, sub-Saharan Africa, risk factors

## Abstract

**Background:**

Dementia is one of the leading non-communicable causes of disability and mortality in older adults, with recent research showing that it is increasing in low-middle-income countries compared to high-income countries. As such, multidisciplinary efforts are needed to effectively reduce the prevalence and risk of dementia through quick screening, diagnosis, and management of those with dementia and those at risk.

**Aim:**

The study’s objectives were to estimate the prevalence of dementia and measure the sociodemographic and clinical risks in older adults in low socioeconomic communities.

**Setting:**

The study was conducted among older adults aged ≥ 60 years from the iLembe district in South Africa.

**Methods:**

This cross-sectional, one-phased, household study was conducted to screen for dementia over 8 months between October 2018 and October 2019. Demographic and clinical data were collected using a semi-structured questionnaire. In addition, the Mini-Mental Status Exam, Ascertain Dementia Eight-item questionnaire and Instrumental Activities of Daily Living Scale were administered to a multi-stage cluster sample of 320 participants to ascertain dementia prevalence. Frequencies and multivariate logistic regression were conducted to determine risk factors correlated with dementia.

**Results:**

The prevalence of dementia was 13.4%. Participants aged 80 years and above were 2.73 times more likely to develop dementia than participants younger than 80 years. Those with an education level of Grade 1–7 had a 69% less chance of developing dementia than those without formal education. Single participants showed an almost seven-fold increase in dementia. Lastly, depression increased the risk of dementia by two-fold.

**Conclusion:**

Dementia was probable in over one-sixth of the sample. Dementia risk factors were both modifiable and non-modifiable.

**Contribution:**

Dementia prevalence in South Africa is increasing and therefore it is crucial to develop a dementia plan that is specific to the South African context which will include strategies for early identification of the disease, reducing modifiable risks and strategic management of dementia associated medical conditions such as depression and vascular diseases.

## Introduction

Dementia is one of the leading causes of disability and mortality in older adults (Alzheimer’s Disease International [ADI] 2015). According to Nichols et al. (2022), more than 60% of people with dementia live in low-middle-income countries (LMICs), with the highest estimates in Asia and Africa (ADI 2015; Nichols et al. 2022). In Africa, the regional forecast of dementia prevalence in people ≥ 60 years ranges from 5.1% in North Africa to 20.0% in sub-Saharan Africa (SSA) (Akinyemi et al. 2022). In South Africa, dementia is reported to be between 6.3% and 12.5% (De Jager et al. 2017; Farina et al. 2023; Ramlall et al. 2013; Vanderpoel, Heyns & 10/66 Dementia Research Group 2012). The prevalence of dementia in LMICs, such as in South Africa, has been predicted to increase, and the increase is associated with low interventions aimed at risk reduction in these countries (ADI 2015; Nichols et al. 2022).

A combination of non-modifiable factors, such as age and gender, and modifiable factors, such as depression, hypertension, education, and socioeconomic levels, are associated with dementia (ADI 2017; George-Carey et al. 2012; Mavrodaris, Powell & Thorogood 2013; Olanyika & Mbuyi 2014; Paddick et al. 2014). In Africa, increasing age continues to be a significant risk factor, with the highest rate of dementia expected from the 70–80+ age group (ADI 2017; George-Carey et al. 2012; Gorelick et al. 2011; Mavrodaris et al. 2013; Olanyika & Mbuyi 2014). In addition, women are considered more at risk than men (ADI 2017; De Jager et al. 2017; Yusuf et al. 2018), because women have a higher life expectancy and increased neurobiological vulnerability during the postmenopausal period (Podcasy et al. 2016). Another risk factor, mainly in LMICs, is related to regional settings. For example, people in urban areas seem less vulnerable to dementia than those in rural areas (De Jager et al. 2015; Gureje et al. 2011). This is because commonly rural populations are older and because of poor access to health, they have higher rates of poorly managed chronic conditions, such as hypertension, diabetes, hyperlipidaemia, and depression compared to urban populations (Olivier, Cacabelos & Naidoo 2018; Rahman et al. 2020). The combination of these risk factors make rural communities disproportionately vulnerable to dementia.

There is a paucity of dementia prevalence and risk studies in Southern Africa; therefore, a need was identified for epidemiological studies in South Africa (De Jager et al. 2015). The authors identified two community-based studies estimating dementia prevalence in South Africa (De Jager et al. 2017; Ramlall et al. 2013; Vanderpoel et al. 2012). The two studies varied in methods used and were in different provinces. One study was conducted in KwaZulu-Natal (KZN) nursing homes, but there were no community studies (Ramlall et al. 2013). Therefore, the authors conducted a community study in one of the 11 districts of KZN province. This study estimated the prevalence of dementia and measured the sociodemographic and clinical risks in older adults in low socioeconomic communities in the iLembe district. It is important to measure dementia prevalence and identify risk factors in people with dementia to develop reduction strategies and improve the longevity of older adults.

## Research methodology

### Study design and setting

A one-phase, cross-sectional community study was conducted in the iLembe district. iLembe is located about 75 km north of Durban, in KZN, South Africa. It is mostly a rural district, with agriculture as the main economic activity. Four local municipalities constitute the district: Mandeni, KwaDukuza, Ndwedwe, and Maphumulo. It comprises four ethnic groups, with a majority of IsiZulu speakers. Most households are low-middle income, earning just the minimum wage of ZAR R3 500.00 per month and dependent on government grants and state health care.

### Study population and sampling strategy

The targeted population for this study was older adults ≥ 60 years. ILembe district has approximately 53 956 older adults (Statistics South Africa 2016). The sample was recruited using multi-stage cluster and simple random sampling techniques. In the first stage, using simple random sampling, the iLembe district was selected from other districts in KZN. In the second stage, eight areas were randomly selected from the four municipalities in the iLembe district. In the third stage, the first household was selected in each area, and subsequent households were determined using a sampling interval of 5. From the population, 320 participants who were ≥ 60 years old were recruited. All participants had to give consent to be included in the study. Participants also needed to have an available caregiver or informant to provide collateral information. This sample type is a participant-informant dyad, and 300 informants were recruited.

#### Inclusion criteria

- For participants: All participants had to be ≥ 60 years old. The participants did not need to be literate but had to be able to see and hear well enough to do the tests.
- For the informants: Informants needed to be family members, caregivers or close neighbours, ≥ 18 years old, who were well-informed of the participants’ daily activities and health status, and could provide collateral information.

### Data collection

#### Procedure

The interviews were conducted in the participants’ homes by well-trained field workers. A semi-structured clinical interview was conducted with both the participants and the informants. The participants were also administered a neuropsychological assessment battery, including the Mini-Mental Status Exam (MMSE). In addition, the informants answered questions about the participants functioning on the Ascertain Dementia Eight-item questionnaire (AD8) and the Instrumental Activities of Daily Living Scale (IADLS). The interviews were conducted in either isiZulu or English, depending on the participants’ preferred language. The interviews with participants and informants took approximately 2 h.

#### Instruments

A clinical questionnaire was used to obtain information regarding biographical details such as gender, age, home language, education, and clinical variables such as medical history, lifestyle behaviours, psycho-social factors, and family history of the participants. The MMSE, AD8 and Lawton’s IADL (Folstein, Folstein & McHugh 1975; Galvin et al. 2005; Lawton & Brody 1969) were administered to measure cognitive decline and changes in daily functioning. The MMSE is a commonly used test that assesses multidomain cognitive functioning (Schutte, Tsikane & Nchoe 2021). The test has 11 items, with a total score of 30 (Creavin et al. 2016). The cut-off score was adjusted for education level on the MMSE – for participants with no formal education, a score of < 21 was used, and < 22 for the rest of the sample, as similarly done in other studies with participants with low levels of education (El-Tallawy et al. 2013; Mao et al. 2018). In this study, the MMSE showed good reliability with a Cronbach’s alpha of 0.703 (*M* 22.80; *SD* 5.06) and a specificity of 76.5%. In addition to the MMSE, the AD8 obtained information from reliable informants. The AD8 helps to discriminate between signs of normal ageing and mild dementia. The AD8 contains eight items that test for memory, orientation, judgement, and function. For AD8, a score > 2 signified cognitive impairment (Galvin et al. 2006). The AD8 has been used in other studies with participants with low education levels (Ong et al. 2021; Yan et al. 2021). The AD8 had a specificity of 54.2%. The IADLS was administered to informants to assess the participants’ independence in performing daily functioning (Lawton & Brody 1969). The summary score ranged from 0 (low function, dependent) to 8 (high function, independent) for women and 0 to 5 for men (Mao et al. 2018). The IADLS had a specificity of 58.5. All three tests had a sensitivity of 100%.

According to the International Classification of Diseases (ICD 11) (World Health Organization [WHO] 2019), dementia is characterised by a decline in cognitive functioning, which must be observable by other people and should affect daily functioning. Therefore, study participants were classified as having dementia if they had cognitive decline based on the MMSE score of < 22, plus cognitive decline reported by caregivers based on the AD8 score of > 2, and decreased daily functioning on the IADLS. Self-report items were used to assess if participants had delirium and any other mental illness that could account for the symptom.

### Data analysis

All information was captured onto hand-held tablet devices using the Mobenzi Researcher software (Mobenzi 2021). Data was exported from the Mobenzi Researcher database to Microsoft Excel.

The statistical data analysis was conducted in R statistical computing software version 3.6.3 of the R Core Team (2021). Dementia was ascertained using the combined scores of the MMSE, AD8 and IADL (Folstein et al. 1975; Galvin et al. 2005; Lawton & Brody 1969) and was reported descriptively. Chi-square and Fischer’s tests measured associations between dementia and sociodemographic factors. Lastly, multivariate logistic regression was used to determine the likelihood of dementia based on the sociodemographic and health variables. Odds ratios (ORs), 95% confidence intervals (CIs) and *p* values (α) were calculated. Statistical significance was set at < 5%.

### Ethical considerations

Ethical clearance was obtained from the University of KwaZulu-Natal’s Human Research Ethics Committee (HSS/1016/017D). Written informed consent was obtained from the participants; where participants could not provide consent, assent was obtained from family members. Necessary steps were taken to preserve participant anonymity and confidentiality. Participants that had major disabilities which affected participation and cognitive decline were not included in the study. After data analysis, participants who screened positive for dementia were informed of the results and referred to their nearest healthcare centre for further assessments. The option of counselling was offered to participants as needed.

## Results

A total of 320 participants were recruited. Of these, some participants were from KwaDukuza (86; 26.9%), KwaMaphumulo (40; 12.5%), eMandeni (128; 40%) and eNdwedwe (66; 20.6%). Most participants were black African (225; 70.3%) and isiZulu speakers (220; 68.8%). The mean age of the participants (*N* = 320) was 69.43 (standard deviation [SD] = 7.76), with a minimum age of 60 years and maximum age of 94 years (Table 1).

**TABLE 1:** Sociodemographic profile of informants and participants.

Demographic variables	Informants (*N* = 300)		Participants (*N* = 320)	
	*n*	%	*n*	%
**Setting**				
Urban	135	45.0	146	45.6
Rural	165	55.0	174	54.4
**Age (years)**				
18–39	160	53.3	-	-
40–59	78	26.0	-	-
60–79	58	19.3	282	88.1
80+	4	1.3	38	11.9
**Ethnicity**				
African person	224	74.7	225	70.3
Coloured person	3	1.0	6	1.9
Indian person	70	23.3	84	26.3
White person	3	1.0	5	1.6
**Sex**				
Male	86	28.7	79	24.7
Female	214	71.3	241	75.3
**Education**				
No formal education	12	4.0	62	19.4
Primary	50	16.7	159	49.7
Secondary	111	37.0	72	22.5
Matric	84	28.0	14	4.4
Tertiary	43	14.0	13	4.0
**Marital status**				
Single	192	64.0	54	16.9
Married	69	23.0	108	33.8
Divorced	7	2.3	10	3.1
Widowed	21	7.0	141	44.0
Cohabitating	11	3.7	7	2.2

### Dementia prevalence

Of the 320 participants, 43 screened positive for dementia, giving a dementia prevalence of 13.4%. Dementia was higher in those from KwaDukuza (14; 32.6%), from rural areas (25; 58.1%), in females (36; 83.7%), those with education levels between the Grades 1 to 7 (21; 48.8%), were widowed or divorced (24; 55.8%), and had a household income of < R3500 (39; 90.7%) (Table 2).

**TABLE 2:** Participants’ characteristics associated with dementia (*N* = 320).

Dementia	No dementia (*N* = 277)†		Dementia (*N* = 43)‡		*X* ^2^
	*n*	%	*n*	%	*p*
**Municipality**	-	-	-	-	0.325
KwaDukuza	72	26.0	14	32.6	-
Mandeni	115	41.5	13	30.2	-
Maphumulo	36	13.0	4	9.3	-
Ndwedwe	54	19.5	12	27.9	-
**Setting**	-	-	-	-	0.594
Urban	128	46.2	18	41.9	-
Rural	149	53.8	25	58.1	-
**Ethnicity**	-	-	-	-	0.284
African person	196	70.8	29	67.4	-
Indian person	70	25.3	14	32.6	-
Other	11	4.0	0	0.0	-
**Sex**	-	-	-	-	0.169
Female	205	74.0	36	83.7	-
Male	72	26.0	7	16.3	-
**Age groups (years)**	-	-	-	-	0.115
60–69	168	60.6	21	48.8	-
70–79	80	28.9	13	30.2	-
80+	29	10.5	9	20.9	-
**Education**	-	-	-	-	< 0.001*
Never attended	43	15.5	19	44.2	-
Grade 1–7	138	49.8	21	48.8	-
Grade 8+	96	34.7	3	7.0	-
**Marital status**	-	-	-	-	0.008*
Married or cohabitating	108	39.0	7	16.3	-
Single	42	15.2	12	27.9	-
Widowed or divorced	127	45.8	24	55.8	-
**Total household income**	-	-	-	-	0.334
R3500 and below	236	85.2	39	90.7	-
R3500 above	41	14.8	4	9.3	-
**Hypertension**	-	-	-	-	0.128
No	97	35.0	10	23.3	-
Yes	180	65.0	33	76.7	-
**Blackouts**	-	-	-	-	0.039*
No	256	92.4	35	81.4	-
Yes	21	7.6	8	18.6	-
**Head injury**	-	-	-	-	1.000
No	258	93.1	40	93.0	-
Yes	19	6.9	3	7.0	-
**Frequent headaches**	-	-	-	-	0.115
No	217	78.3	29	67.4	-
Yes	60	21.7	14	32.6	-
**Angina**	-	-	-	-	0.301
No	262	94.6	39	90.7	-
Yes	15	5.4	4	9.3	-
**High cholesterol**	-	-	-	-	0.361
No	217	78.3	31	72.1	-
Yes	60	21.7	12	27.9	-
**Stroke**	-	-	-	-	0.684
No	189	68.2	28	65.1	-
Yes	88	31.8	15	34.9	-
**Diabetes Mellitus**	-	-	-	-	0.088
No	191	69.0	24	55.8	-
Yes	86	31.0	19	44.2	-
**Epilepsy**	-	-	-	-	0.352
No	275	99.3	42	97.7	-
Yes	2	0.7	1	2.3	-
**Depression**	-	-	-	-	0.018*
No	250	90.3	33	76.7	-
Yes	27	9.7	10	23.3	-
**Alcohol use**	-	-	-	-	0.448
No	213	76.9	33	76.7	-
Used to but stopped	30	10.8	7	16.3	-
Yes	34	12.3	3	7.0	-
**Tobacco use**	-	-	-	-	0.849
No	223	80.5	36	83.7	-
Used to but stopped	20	7.2	3	7.0	-
Yes	34	12.3	4	9.3	-

* , 0.05 level (2-tailed);

** , 0.01 level (2-tailed).

† , 86.6%;

‡ , 13.4%.

Dementia was significantly associated with education, marital status, depression and experiencing blackouts, defined as momentary loss of consciousness (Table 2). Regarding sociodemographic factors, dementia was more prevalent in females, those from areas considered to be rural, those within the age group of 60–69, with level of education between Grade 1 and 7, widowed and those with an income of R3500 and below. Dementia was more prevalent in those with hypertension and depression.

### Dementia risk factors

Table 3 shows that factors associated with dementia in this group of participants included age, education, marital status, and depression.

**TABLE 3:** Multivariate regression analysis of factors associated with dementia.

Explanatory	Unadj†			FullAdj‡			Backstep§		
	OR	CI	α	OR	CI	α	OR	CI	α
**Setting**									
Urban^R^	-	-	-	-	-	-	-	-	-
Rural	1.35	0.70–2.69	0.379	1.84	0.62–5.36	0.263	1.93	0.90–4.27	0.095
**Sex**									
Female^R^	-	-	-	-	-	-	-	-	-
Male	0.58	0.23–1.30	0.218	1.38	0.35–4.97	0.627	-	-	-
**Age group (years)**									
60–69^R^	-	-	-	-	-	-	-	-	-
70–79	1.43	0.66–3.01	0.354	1.34	0.51–3.45	0.544	-	-	-
80+	2.73	1.08–6.50	0.026*	1.61	0.49–5.21	0.426	-	-	-
**Education**									
No formal education^R^	-	-	-	-	-	-	-	-	-
Grade 1–7	0.32	0.16–0.66	0.002*	0.29	0.12–0.70	0.006*	0.31	0.14–0.66	0.003*
Grade 8+	0.05	0.01–0.17	< 0.001**	0.05	0.01–0.21	< 0.001**	0.05	0.01–0.18	< 0.001**
**Marital status**									
Married or cohabitating^R^	-	-	-	-	-	-	-	-	-
Single	5.14	1.87–15.61	0.002*	13.28	3.46–60.59	<0.001**	6.92	2.25–23.78	0.001*
Widowed or divorced	3.29	1.37–9.16	0.013*	4.16	1.26–15.74	0.025*	2.92	1.12–8.66	0.037*
**Household income**									
R3500 and below^R^	-	-	-	-	-	-	-	-	-
R3500 above	0.45	0.11–1.33	0.203	1.27	0.24–5.14	0.750	-	-	-
**Hypertension**									
No^R^	-	-	-	-	-	-	-	-	-
Yes	1.93	0.92–4.44	0.099	2.32	0.82–7.07	0.124	-	-	-
**Head injury**									
No^R^	-	-	-	-	-	-	-	-	-
Yes	1.13	0.26–3.55	0.849	0.83	0.15–3.43	0.805	-	-	-
**Migraines**									
No^R^	-	-	-	-	-	-	-	-	-
Yes	1.91	0.92–3.82	0.073	1.10	0.42–2.78	0.847	-	-	-
**Blackouts**									
No^R^	-	-	-	-	-	-	-	-	-
Yes	2.50	0.93–6.08	0.053	2.56	0.70–9.06	0.146	-	-	-
**Cardiovascular**									
No^R^	-	-	-	-	-	-	-	-	-
Yes	1.37	0.31–4.41	0.628	0.33	0.05–1.66	0.204	-	-	-
**Stroke**									
No^R^	-	-	-	-	-	-	-	-	-
Yes	1.25	0.62–2.46	0.518	1.90	0.77–4.68	0.160	-	-	-
**High cholesterol**									
No^R^	-	-	-	-	-	-	-	-	-
Yes	1.49	0.69–3.03	0.285	1.02	0.35–2.81	0.977	-	-	-
**Diabetes Mellitus**									
No^R^	-	-	-	-	-	-	-	-	-
Yes	1.73	0.88–3.36	0.108	2.04	0.83–5.09	0.121	1.98	0.91–4.36	0.086
**Depression**									
No^R^	-	-	-	-	-	-	-	-	-
Yes	2.70	1.11–6.12	0.021*	3.48	1.01–11.89	0.046*	2.74	1.01–7.11	0.041*
**Drink alcohol**									
No^R^	-	-	-	-	-	-	-	-	-
Used to but stopped	1.66	0.63–3.94	0.274	3.25	0.71–14.69	0.123	-	-	-
Yes	0.61	0.14–1.82	0.429	0.95	0.14–5.37	0.957	-	-	-
**Tobacco use**									
No^R^	-	-	-	-	-	-	-	-	-
Used to but stopped	1.04	0.23–3.25	0.957	1.78	0.20–13.76	0.590	-	-	-
Yes	0.77	0.22–2.09	0.643	2.52	0.45–12.13	0.264	-	-	-
**Family health risks**									
**Heart condition**									
No^R^	-	-	-	-	-	-	-	-	-
Yes	0.81	0.37–1.65	0.575	0.45	0.14–1.31	0.154	-		
**Hypertension**									
No^R^	-	-	-	-	-	-	-	-	-
Yes	0.71	0.36–1.36	0.304	0.92	0.35–2.40	0.864	-		
**Diabetes Mellitus**									
No^R^	-	-	-	-	-	-	-	-	-
Yes	0.76	0.38–1.48	0.427	0.83	0.32–2.13	0.695	-		
**Parkinson disease**									
No^R^	-	-	-	-	-	-	-	-	-
Yes	0.76	0.18–2.29	0.664	0.58	0.11–2.34	0.481	-		
**Dementia**									
No^R^	-	-	-	-	-	-	-	-	-
Yes	1.41	0.50–3.42	0.480	1.60	0.40–5.81	0.484	-	-	-

OR, odds ratio; CI, confidence interval; *^R^*, reference group.

† , risk between dementia and other variables entered simultaneously;

‡ , risk of dementia with adjustment for confounding variable;

§ , risk of dementia with variables entered separately.

* , 0.05 level (2-tailed);

** , 0.01 level (2-tailed).

In the unadjusted model, participants aged 80 years and above were 2.73 times more likely to develop dementia (CI = 1.08–6.50, *p* = 0.026) than participants younger than 80. In the backstep model, those with an education level of Grade 1–7 had a 69% less chance of developing dementia (OR: 0.31; *p* = 0.003) than those without formal education. Compared to married participants or those with a partner, single participants showed an almost seven-fold increase in dementia (OR: 6.92; *p* = 0.001) and nearly three-fold for widowed participants (OR: 2.92; *p* = 0.037). Lastly, depression increased the risk of dementia by two-fold (OR: 2.74; *p* = 0.041).

## Discussion

This study investigated the prevalence and correlates of dementia in older adults in a community setting. The prevalence of dementia was 13.4%. This prevalence rate is a little higher compared to other community studies in South Africa, which reported a prevalence of 6.3% – 12.5% in older adults living in rural and urban settings (Farina et al. 2023; Vanderpoel et al. 2012). It is also a little higher when compared to other studies from other African and Western countries (ADI 2015; Guerchet et al. 2009, 2010, 2014; Gureje et al. 2011; Paraïso et al. 2011; Ogunniyi et al. 2016; Longdon et al. 2012; Yusuf et al. 2011). The different life expectancy rates and differences in research methods are some of the factors that might contribute to variable prevalent rates in some of the studies when compared to this study. With increasing life expectancy in LMICs, it is expected that dementia prevalence will increase (Nichols et al. 2022). Hence, it is anticipated that dementia in South Africa might also increase. However, as seen in the dementia trends in high-income countries (Nichols et al. 2022), dementia increase in LMICs can also be lowered if priority is placed on reducing modifiable risk factors.

Risk factors associated with dementia in this study included ageing, depression, not having a partner and education. Low education, depression and loneliness have also been identified in other African studies (George-Carey et al. 2012; Mavrodaris et al. 2013; Olanyika & Mbuyi 2014; Paddick et al. 2014; Yusuf et al. 2018), confirming the prominent plight of these factors in older African populations. Not having a partner in this study increased the risk of dementia by seven-fold for those who are single and three-fold for those who are widowed. This speaks to issues of loneliness and isolation in older adults. Other factors associated with loneliness and isolation in older adults include living alone, losing family or friends, chronic illness, and hearing loss (National Academies of Sciences, Engineering, and Medicine 2020). These results suggest that it is important that older people are included in community activities to combat loneliness and isolation.

Ageing is a primary risk factor for dementia (ADI 2015; De Jager et al. 2017; Longdon et al. 2012), doubling the risk of dementia in those aged < 80 years in this study compared to those aged 60 years – 69 years. Alzheimer’s Disease International (2015) reported that a person’s risk increases as they age, doubling every 5 years. In addition, older people are more likely to live with other comorbid illnesses such as hypertension, stroke, diabetes, and hypercholesterolaemia, also associated with dementia (Guo et al. 2018; Huang et al. 2015), thereby increasing the vulnerability of an older person to developing dementia.

Identifying and reducing risks, such as increasing the quality and levels of education and improving the control of depression and cardiovascular diseases, can effectively lower the incidence of dementia (ADI 2015; Nichols et al. 2022; WHO 2017). Quality education and cognitive stimulation programmes must be prioritised in developing countries to build and enhance cognitive reserve from a younger age. Further, public health interventions should be prioritised at a grassroots level to educate people about dementia risk factors such as cardiovascular risks. Addressing risk factors through public health interventions is a pathway to reducing dementia prevalence and altering the trajectory of age-specific prevalence. Lastly, the treatment gap between people diagnosed with dementia and those undiagnosed but with dementia must be reduced. This means that public health screenings for dementia should be encouraged. Older people should be screened at community clinics when they routinely visit to collect medications. To achieve this, primary healthcare staff can easily administer assessment tests that are readily available, easy to administer, and not time-consuming. There is a greater need for using validated and simple neuropsychological tools for early diagnosis of cognitive impairment (Ramlall et al. 2013). These tests must be easily incorporated into the routine screening of older people in primary healthcare settings in conjunction with the DSM-5 and/or the ICD-11. Cases of dementia can be identified, and routine care commenced, including laboratory testing and symptomatic management.

## Conclusion

This study’s primary objective was to measure the risk factors and prevalence of dementia in the iLembe district. Dementia prevalence was 13.4%. Many people with dementia in South African communities likely remain undiagnosed, and do not have access to treatment, care and support that a formal diagnosis can provide. More longitudinal studies need to be done to ascertain the actual trajectory of dementia in South Africa. The identified risk factors for dementia were age, low levels of education, lack of a spouse and a history of depression. The early identification of cognitive impairment is important in devising possible interventions that can help to reduce the burden on those affected and impacted by dementia and to lighten the socioeconomic burden of the disease on the country’s resources (Schutte et al. 2021).

Future research in South Africa needs to be more representative and include longitudinal studies. In addition, future research needs to focus on exploring and eliminating the modifiable risk factors mentioned for populations identified as being at risk, especially those in rural areas and low-income households. It is recommended that when tests such as the MMSE are used to screen for dementia, they are used with caution and in combination with other tests. This study used a combination of tests and only counted participants as having dementia if the combined test results corresponded with the Diagnostic and Statistical Manual of Mental Disorders (DSM) 5 and ICD 11 criteria for dementia instead of relying on the results of the individual psychometric tests. Combining the MMSE, AD8 and IADL provides better diagnostic accuracy in the absence of laboratory tests, and can be easily incorporated into routine care by primary healthcare providers for diagnosing dementia.

Limitations of this study include the cross-sectional design, which lessened the ability to establish causal-effect relationships between dementia and the independent variables. In addition, this study did not include laboratory testing. However, other studies have predicted dementia without laboratory testing, and those screened as positive for dementia were referred for further testing at their healthcare institutions. This study was conducted in one district and was not a national study. Therefore, results should be generalised with caution to other communities in South Africa. Notwithstanding these limitations, the study provides insight relevant to issues related to dementia prevalence in South Africa and other developing countries.
